# Lamivudine sensitivity - a case report

**DOI:** 10.1186/1471-2334-14-S3-E39

**Published:** 2014-05-27

**Authors:** Leelavathy Budamakuntla, Prabhakar Basappa, Vidya Shankar, Nirmala, Ravi Kumar, Shwetha Suryanarayana

**Affiliations:** 1 Department of Dermatology, Bowring & Lady Curzon Hospital, Bangalore Medical College& Research Institute, Bangalore, Karnataka, India; 2 Department of Medicine, Centre of Excellence for ART, Bowring & Lady Curzon Hospital, Bangalore Medical College & Research Institute, Bangalore, Karnataka, India

## Background

Lamivudine is a dideoxy nucleoside analogue – NRTI, widely used in the treatment of HIV infection. It is a best tolerated drug with long term safety profile and most preferred in all regimens. Skin hypersensitivity reactions to lamivudine are rare and reported to be around 9%.

## Case report

A 29 year old HIV positive female was initiated on ART on 5/11/2012 with first line regimen, (TDF+3TC+NVP) as patient was anaemic. Patient came back with Nevirapine rash and ART was stopped on 20/12/12. Nevirapine was substituted with Efavirenz after rashes healed on 2/1/13. Patient developed severe itching with rashes just after three doses and hence ART was stopped. After one month, she was counseled, hospitalized and was rechallenged with only TDF+3TC; patient again developed severe itching with only one dose. Hence, patient was challenged with only lamivudine. With just one dose of lamivudine, patient developed hypersensitivity reaction within few hours with facial oedema, redness, tightness of skin with maculopapular rash and generalized blanchable erythema, giddiness, and hypotension and collapsed. She was resuscitated with dopamine, IV fluids and antihistamines.

## Conclusion

Nucleoside reverse transcriptase inhibitors (NRTIs) such as lamivudine can cause skin rash in 1-10% of patients. Lamivudine related cutaneous side effects are probably under estimated. Lamivudine hitherto considered a safe drug can cause serious side effects in some patients as reported here. Hence, careful observation and timely intervention is mandatory to avoid fatalities in those patients receiving NRTI based antiretroviral therapy.

